# 基于高效液相色谱-串联质谱测定孕妇血清中7种全氟和多氟化合物及新生儿神经行为评价

**DOI:** 10.3724/SP.J.1123.2023.07022

**Published:** 2024-02-08

**Authors:** Zihao WANG, Fengzhi LIANG, Xuerong CHEN, Ping WU, Wei WU

**Affiliations:** 湖北中医药大学检验学院, 湖北 武汉 430065; School of Laboratory Medicine, Hubei University of Chinese Medicine, Wuhan 430065, China

**Keywords:** 高效液相色谱-串联质谱, 全氟和多氟化合物, 血清, 孕期暴露, 神经行为发育, high performance liquid chromatography-tandem mass spectrometry (HPLC-MS/MS), perfluoroalkyl and polyfluoroalkyl substances (PFASs), serum, pregnancy exposure, neurobehavioral development

## Abstract

以同位素为内标,建立了高效液相色谱-串联质谱测定孕妇血清中7种全氟和多氟化合物(PFASs)的方法,同时评估新生儿的神经发育水平,探讨PFASs对新生儿神经行为发育的影响。本研究依托鄂州市妇幼保健院建立的出生队列,采集孕妇孕晚期血清样本,并放置于-80 ℃冰箱后统一检测。首先将血清样品解冻,之后依次加入^13^C标记的内标物、碳酸钠-碳酸氢钠缓冲液和四丁基硫酸氢铵溶液(TBAS),在超声处理后加入甲基叔丁基醚(MTBE)洗脱两次,收集所有洗脱液,涡旋混匀后离心,转移上清液至进样瓶中,随后进行检测。使用新生儿神经行为测定法(neonatal behavioral neurological assessment, NBNA)对新生儿的神经行为进行评分,评估孕期PFASs暴露对新生儿神经行为发育的影响。结果显示,7种PFASs在0.1~200 ng/mL内线性关系良好,检出限(LOD)为0.006~0.020 ng/mL,定量限(LOQ)为0.020~0.066 ng/mL,加标回收率为84.6%~116.8%。该方法所需样本量少,具有较高的准确度和精密度。实际样品检测发现,456名孕妇血清中7种PFASs的检出率均≥97.4%,其中暴露水平最高的是全氟辛酸(PFOA),中位数水平为19.4 ng/mL。同时,对379名新生儿的神经行为进行评估,线性回归模型表明,孕期母体体内的全氟辛烷磺酸(PFOS)每增加一个自然对数(ln)水平,新生儿的主动肌张力及一般反应分别降低0.36分(95%置信区间(CI): -0.64~-0.08)和0.34分(95% CI: -0.61~-0.07);全氟壬酸(PFNA)每增加一个ln水平,新生儿的被动肌张力和NBNA总分分别降低0.38分(95%CI: -0.74~-0.01)和0.37分(95% CI: -0.68~-0.06);全氟癸酸(PFDA)每增加一个ln水平,新生儿的行为能力降低0.28分(95% CI: -0.54~-0.01);而全氟己烷磺酸(PFHxS)每增加一个ln水平,新生儿的NBNA总分上升0.27分(95% CI: 0.05~0.48)。性别分层分析发现,男性新生儿对PFASs的神经毒性作用更加敏感。通过此方法测定孕妇血清中的7种PFASs,并结合新生儿的神经行为评估发现,孕期PFASs暴露会影响新生儿的神经行为发育。

## Abstract (en)

Perfluoroalkyl and polyfluoroalkyl substances (PFASs) have been extensively used as synthetic fluorine-containing compounds in various consumer products, including surfactants, cookware, lubricants, clothing, and food packaging, since the 1950s. Evidence has shown that PFASs cross the placental barrier and interfere with fetal thyroid hormone homeostasis, which is crucial for fetal growth and neurobehavioral development in children aged 2-9 years. However, no epidemiological data on the association between prenatal PFAS exposure and neonatal neurobehavioral development are available.

In this study, we explored the association between prenatal PFAS exposure and neonatal neurobehavioral development based on the Ezhou cohort study. Blood samples (10 mL) were collected during the third trimester of pregnancy (28-36 weeks) at the Ezhou maternal and child health hospital. The blood specimens were centrifuged at 4000 r/min for 15 min immediately after collection, separated, stored at -80 ℃. The samples were analyzed for seven PFASs, namely, perfluorooctanoic acid (PFOA), perfluorooctane sulfonate (PFOS), perfluorohexane sulfonate (PFHxS), perfluorononanoic acid (PFNA), perfluorodecanoic acid (PFDA), perfluoroheptanesulfonic acid (PFHpS), and perfluorooctane sulfonamide (PFOSA). The PFASs were separated using a C_18_ column (100 mm×2.1 mm, 1.7 μm) at an oven temperature of 40 ℃, injection volume of 10 μL, and flow rate of 0.4 mL/min via gradient elution with methanol and ammonium acetate aqueous solution. The instrument was operated in negative electrospray ionization mode with multiple reaction monitoring. The correlation coefficients (*r*^2^), limits of detection (LODs) and quantification (LOQs), and spiked recoveries of the seven PFASs were 0.993-0.999, 0.006-0.020 ng/mL, 0.020-0.066 ng/mL, and 84.6%-116.8%, respectively. Neonatal behavioral neurological assessment (NBNA) was used to evaluate newborn cognitive development 72 h after birth; this tool consisted of five clusters, including behavior (six items), passive muscle tone (four items), active muscle tone (four items), primitive reflexes (three items), and general assessment (three items). Each item was rated on a three-point scale (0, 1, or 2), with the 20 items having a maximum score of 40. A total of 379 mother-newborn pairs were included in the analysis. The PFASs with the highest exposure levels was PFOA, with median levels of 19.4 ng/mL. Linear regression models were used to test the effects of ln-converted PFAS levels in newborns. After adjusting for confounding factors, the linear regression model showed that PFOS exposure during pregnancy was associated with decreased active muscle tone(*β*(95% CI): 0.36(-0.64, 0.08)) and general assessment(*β*(95% CI): 0.34(-0.61, 0.07)) in all newborns. Furthermore, PFNA exposure was associated with decreased passive muscle tone(*β*(95% CI): 0.38(-0.74, 0.01)) and total NBNA(*β*(95% CI): 0.37(-0.68, 0.06)). PFDA exposure was associated with decreased behavior(*β*(95% CI): 0.28(-0.54, 0.01)), while PFHxS exposure was associated with elevated total NBNA(*β*(95% CI): 0.27(0.05-0.48)). Gender stratification analysis showed that PFOS exposure during pregnancy was associated with decreased active muscle tone(*β*(95% CI): 0.54(-0.73, 0.35)) and general assessment(*β*(95% CI): 0.50(-0.88, 0.13)), PFNA exposure during pregnancy was associated with decreased passive muscle tone(*β*(95% CI): 0.67(-1.2, 0.14)) and total NBNA(*β*(95% CI): 0.45(-0.91, 0.01)), PFDA exposure during pregnancy was associated with decreased behavior(*β*(95% CI): 0.44(-0.71, 0.17)), PFHxS exposure was associated with elevated total NBNA(*β*(95% CI): 0.41(0.02-0.80)) in male newborns, and PFOA exposure was associated with decreased general assessment(*β*(95% CI): -0.27(-0.51, 0.02)), and PFDA exposure was associated with elevated behavior(*β*(95% CI): 0.46(0.40-0.52)) in female newborns. The proposed method separates and detects various PFASs without the need for cumbersome pretreatment processes, and has the advantages of low LODs, satisfactory recoveries, and accurate precision. Thus, it allows for the simultaneous analysis of trace PFASs in microserum samples from pregnant women. Our results also showed that prenatal PFAS exposure can lead to neurobehavioral disorders in offspring, with male newborns showing greater sensitivity than female newborns.

全氟和多氟化合物(PFASs)是一类新兴的环境内分泌干扰物,常被用于表面活性剂^[1]^、洗涤剂^[2]^、防火泡沫^[3]^、皮革制品和食品包装的制造等^[4]^。PFASs的半衰期很长,其从产品中释放后可广泛分布于空气、水、土壤中^[5⇓-7]^。除此之外,PFASs也广泛存在于各种食品中(如蔬菜、乳制品、饮料、鸡蛋、肉类制品、鱼类和贝类),而饮食摄入也是人类接触PFASs的另一重要途径^[8⇓-10]^。由于PFASs中的C-F键具有较强的稳定性,PFASs表现出极强的持久性,并具有生物蓄积性和毒性^[11]^。研究表明,PFASs在人体血液和肝脏中所检测到的含量最高^[12,13]^。血清蛋白是PFASs结合的主要目标物,这可能是因为PFASs具有生物富集性^[14]^。然而,目前PFASs的检测基质大多局限于水、食品、纺织品等,有关血液样品中PFASs的检测方法较少。因此,建立血液中多种PFASs的快速、准确定量检测方法,对监测人群PFASs的暴露水平具有重要意义。

孕妇作为特殊人群,其宫内遭遇的环境应激可以决定子代出生后的疾病风险^[9,15]^。日本的一项研究表明,孕妇体内全氟壬酸(PFNA)的含量与新生儿的体长呈负相关^[16]^。上海市疾病预防控制中心针对本辖区的一项调查发现,孕期全氟辛酸(PFOA)暴露会干扰孕妇体内的甲状腺水平,且孕妇体内的PFOA含量与新生儿的出生体重呈负相关^[17]^。陈晨春^[18]^通过调查1000多对广西地区的孕产妇与新生儿发现,孕期全氟辛烷磺酸(PFOS)的暴露水平与胎儿宫内生长受限(intrauterine growth restriction, IUGR)呈正相关。除此之外,有关孕期PFASs暴露与新生儿神经行为发育影响的研究较少。

针对以上问题,本课题组建立了基于高效液相色谱-串联质谱(HPLC-MS/MS)检测孕妇血清中7种(PFASs)的分析方法。该方法采用液液萃取作为样品前处理方法,使用内标法定量。依托于已经建立的出生队列,将该法应用于孕妇血清中PFASs的监测,追踪并评估新生儿的神经行为发育,分析孕期PFASs暴露对新生儿神经发育的影响。

## 1 实验部分

### 1.1 仪器、试剂与材料

Xevo TQ-S Micro高效液相色谱-质谱联用仪(美国Waters公司); XH-B涡旋混匀器(江苏康健医疗用品有限公司); KQ-500VDE数控超声波清洗器(昆山市超声仪器有限公司); H2050R冷冻离心机(湖南湘仪实验室仪器开发有限公司); QYN100-1水浴氮吹仪(武汉泰仕德科技有限公司)。

超纯水仪(美国Millipore公司);甲醇、甲酸(均为HPLC级,美国Thermo Fisher公司);甲基叔丁基醚(MTBE)、四丁基硫酸氢铵(TBAS)(均为HPLC级,美国Sigma-Aldrich公司);碳酸钠(纯度≥99.5%)、碳酸氢钠(纯度≥99.8%)和乙酸铵(纯度≥99.0%)(国药集团)。

7种PFASs标准品和3种同位素内标单标溶液均购于加拿大威灵顿实验室。7种PFASs标准品:PFOA、PFOS、PFNA、全氟癸酸(PFDA)、全氟己烷磺酸(PFHxS)、全氟庚基磺酸(PFHpS)、全氟辛烷磺酰胺(PFOSA),溶剂均为甲醇,质量浓度均为5 μg/mL; 3种同位素内标单标溶液:^13^C_4_-PFOS、^13^C_4_-PFOA、^13^C_4_-PFHxS,溶剂均为甲醇,质量浓度均为2 μg/mL。其中,PFOSA、PFHxS以^13^C_4_-PFHxS为内标,PFHpS、PFOA以^13^C_4_-PFOA为内标,PFDA、PFOS和PFNA以^13^C_4_-PFOS为内标。

### 1.2 标准溶液的配制

混合标准工作溶液:分别吸取200 μL PFASs标准品(5 μg/mL)至同一5 mL容量瓶中,用甲醇定容,配制成质量浓度为200 ng/mL的混合储备液,于4 ℃保存待用。用甲醇对混合储备液进行逐级稀释,配制成系列质量浓度(0.1、0.5、2、10、50、100、200 ng/mL)的混合标准工作溶液,并于4 ℃保存。

混合内标工作液:先分别吸取500 μL的3种同位素内标单标溶液至同一10 mL容量瓶中,用甲醇定容至10 mL,配制成质量浓度为100 ng/mL的内标储备液;再用甲醇依次稀释成质量浓度为1.0、10、50 ng/mL的混合内标工作液,并分装为1 mL/支,于-20 ℃保存待用。

### 1.3 问卷调查及样本采集

本课题基于湖北省鄂州市妇幼保健院建立的出生队列展开研究,该队列以2019年招募的孕妇作为研究对象;在孕妇首次产检时,由护士对孕妇进行面对面问卷调查,以获得孕产妇的社会人口学信息。收集孕妇在孕晚期时(28~36周)的血液样本(10 mL),以4000 r/min离心15 min,之后取出上层血清,置于-20 ℃冰箱中冻存,然后转入-80 ℃冰箱中长期保存。样本采集期间共收集456名孕妇孕晚期的血清样本。随访期间,排除流产、引产、信息不完整及子代失访者等情况,共有379名新生儿完成了新生儿神经行为评估。本研究已得到湖北中医药大学伦理委员会批准(No. 2021-IEC-015),招募志愿者均已签署知情同意书。

### 1.4 样品前处理

将采集的血清样本从-80 ℃冰箱中取出,放置在4 ℃冰箱中过夜,充分解冻后再转移至室温。先将5 μL混合内标工作液加入至15 mL聚丙烯离心管中,然后再依次加入200 μL血清样品、2 mL碳酸钠-碳酸氢钠缓冲液(pH 9.2)、1 mL TBAS,超声处理20 min;之后向离心管中加入4 mL MTBE,置于涡旋混匀器上充分振荡2 min,然后超声20 min,以4000 r/min离心15 min。用聚丙烯材质吸管将有机相上清液转移至新的离心管中,向原离心管中再次加入4 mL MTBE,重复上述操作,合并两次上清液,于50 ℃恒温氮吹至干,再加入200 μL甲醇复溶,置于涡旋混匀器上充分振荡2 min,以4000 r/min离心15 min,最后将上清液转移至进样小瓶中直接用于上机检测。

### 1.5 分析条件

色谱柱:Waters ACQUITY HPLC BEH C_18_柱(100 mm×2.1 mm, 1.7 μm);流动相:A相为甲醇,B相为2 mmol/L乙酸铵水溶液。梯度洗脱程序:0~5 min, 60%A; 5~10 min, 60%A~80%A; 10~12 min, 80%A~90%A; 12~15 min, 90%A; 15~20 min, 90%A~10%A。流速0.4 mL/min;进样体积10 μL;柱温40 ℃;样品室温度10 ℃。

离子源:电喷雾电离(ESI)源,负电离模式;扫描模式:多反应监测(MRM)模式;毛细管电压:0.5 kV;离子源温度:150 ℃;脱溶剂气温度:550 ℃;脱溶剂气流量:1000 L/h;碰撞气:氩气。其他质谱参数见表1。

**表1:** 7种PFASs及3种同位素内标的质谱参数

Compound	Retention time/min	Parent ion^*^(*m/z*)	Daughter ion (*m/z*)	DP/V	CEs/eV
Perfluorooctane sulfonamide (PFOSA)	3.85	402.8	83.9	-98	-79, -90
Perfluorohexane sulfonic acid (PFHxS)	4.18	398.9	80.1	-130	-40, -50
Perfluoroheptane sulfonic acid (PFHpS)	4.50	448.9	80.1	-60	-2, -18
Perfluorooctanoic acid (PFOA)	4.76	412.9	368.9	-60	-5, -16
Perfluorodecanoic acid (PFDA)	5.12	512.9	468.9	-75	-5, -14
Perfluorooctane sulfonate (PFOS)	5.38	498.9	99.0	-150	-50, -66
Perfluorononanoic acid (PFNA)	5.75	462.9	418.9	-65	-5, -14
^13^C_4_-PFHxS	4.20	401.1	79.8	-135	-40, -50
^13^C_4_-PFOA	4.76	412.9	371.9	-70	-7, -16
^13^C_4_-PFOS	5.38	503.1	99.0	-170	-50, -66

* Quantitative ion; DP: declustering potential; CE: collision energy.

### 1.6 研究对象基本信息

纳入此次研究的共有379对孕产妇及其子代新生儿,其中男性新生儿的比例(52.8%)和体重((3423.4±452.8) g)均略高于女性新生儿(47.2%, (3326.4±602.8) g)。孕妇的平均孕龄为27.3岁,孕产妇的居住地区以城区为主(77.5%),学历大多为高中(37.2%)和大专(39.1%),家庭年收入以10万~20万居多(46.4%),且大多数孕妇为头胎妊娠(67.5%),居住房屋建筑类型主要为钢筋混凝土(61.0%),人均住房面积为10~20 m^2^(44.9%),相关信息见表2。

**表2:** 孕妇的人口学特征

Characteristic	Number of pregnant women
Living zone	
City/Town	294
Country	85
Mother’s schooling time^*^	
< 9	90
9-12	141
>12	148
Annual household income/RMB	
<100000	83
100000-200000	175
>200000	121
Parity	
0 (primiparous)	257
≥1 (multiparous)	122
Residential building type	
Reinforced concrete	231
Brick-wood	111
Adobe house	37
Average living space/m^2^	
<10	90
10-20	170
>20	119
Age/years (Mean±SD)^#^	27.3±3.8
Pre-pregnancy BMI/(kg/m^2^)(Mean±SD)^#^	21.6±2.7
Gestational age/weeks (Mean±SD)^#^	39.5±1.4

* Start counting in elementary school; BMI: body mass index; # *n*=379.

### 1.7 新生儿神经行为评价

依据北京协和医院鲍秀兰教授修订的20项新生儿神经行为测定法(neonatal behavioral neurological assessment, NBNA)^[19]^,于孕妇分娩第3天(即新生儿出生72 h内),由儿科医师对新生儿的神经行为进行评估。NBNA的评估内容包括行为能力(6项)、被动肌张力(4项)、主动肌张力(4项)、原始反射(3项)和一般反应(3项),共20项指标,每项指标有3个评分水平(0、1、2分),NBNA满分为40分,本实验中共有379名新生儿完成了神经行为评价。

### 1.8 实验室样本质量控制

样本由统一的调查员专职采集并登记,运输过程中确保无污染。整理问卷及实验室检测数据,采用EpiData 3.1软件建立数据库,以平行双盲的方式录入数据,并对数据进行一致性检验和详细的逻辑纠错。实验试剂均为HPLC级或MS级试剂;前处理过程中使用的滴管、离心管等均为一次性材料,以避免PFASs污染。

## 2 结果与讨论

### 2.1 质谱条件的优化

按照1.5节分析条件,对待测物的母离子、子离子和碰撞能量等参数进行优化。先通过HPLC分离和一级质谱全扫描获得每种待测物的保留时间和母离子,然后优化碰撞能量获得子离子,最后采用MRM模式对待测物进行定性和定量分析。7种PFASs及3种同位素内标的总离子流色谱图见图1,相应的质谱参数见表1。

**图1:**
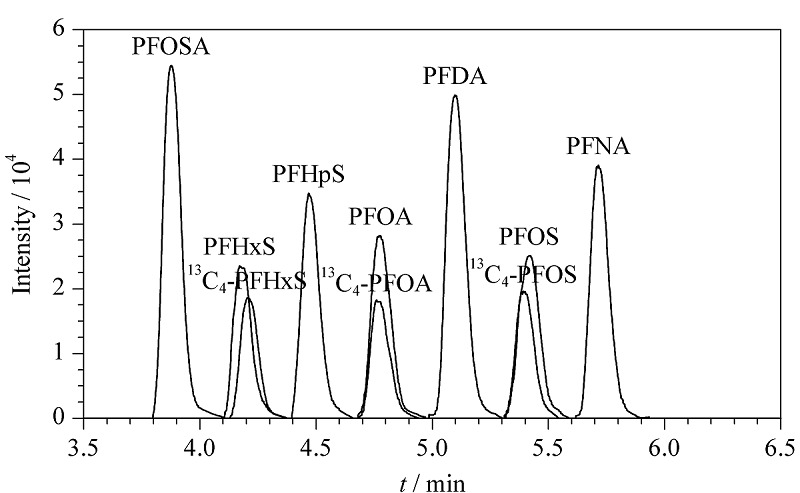
7种PFASs及3种同位素内标的总离子流色谱图

### 2.2 方法学评价

#### 2.2.1 线性范围、检出限与定量限

配制质量浓度为0.1、0.5、2、10、50、100、200 ng/mL的系列混合标准工作溶液并进样分析,以目标物与对应内标的定量离子峰面积之比为纵坐标(*y*)、目标物的质量浓度为横坐标(x,ng/mL),绘制标准曲线。结果如表3所示,7种PFASs在0.1~200 ng/mL内线性关系良好,相关系数(*r*^2^)均≥0.993;以3倍信噪比计算方法的检出限(LOD)、10倍信噪比计算定量限(LOQ), 7种PFASs的LOD为0.006~0.020 ng/mL, LOQ为0.020~0.066 ng/mL。

**表3:** 7种PFASs的线性范围、回归方程、相关系数、检出限和定量限

Compound	Linear range/(ng/mL)	Regression equation	*r*^2^	LOD/(ng/mL)	LOQ/(ng/mL)
PFOA	0.1-200	y=1.023x+0.085	0.998	0.020	0.066
PFOS	0.1-200	y=0.066x+0.025	0.999	0.010	0.033
PFNA	0.1-200	y=0.108x+0.053	0.999	0.012	0.040
PFDA	0.1-200	y=0.025x-0.022	0.998	0.008	0.026
PFHxS	0.1-200	y=0.265x-0.041	0.997	0.010	0.033
PFHpS	0.1-200	y=0.294x-0.028	0.995	0.006	0.020
FPOSA	0.1-200	y=0.038x-0.017	0.993	0.016	0.053

*y*: peak area ratio of the target to the internal standard; *x*: mass concentration, ng/mL.

#### 2.2.2 回收率和精密度

在空白胎牛血清中分别添加低、中、高3个水平(1、10、50 ng/mL)的7种PFASs,进行加标回收试验,每个加标水平进行6次平行试验,并计算加标回收率和相对标准偏差(RSD)。如表4所示,7种PFASs在3个加标水平下的回收率分别为84.6%~116.8%、85.7%~112.5%和84.6%~107.8%,对应的RSD分别为5.8%~16.8%、3.8%~18.2%和6.7%~18.0%。实验结果说明,该方法准确可靠,能够用于实际样品的检测。

**表4:** 7种PFASs在胎牛血清中的加标回收率和相对标准偏差(*n*=6)

Compound	1 ng/mL			10 ng/mL			50 ng/mL	
	Recovery/%	RSD/%		Recovery/%	RSD/%		Recovery/%	RSD/%
PFOA	114.0	9.5		108.8	6.8		94.7	7.8
PFOS	112.3	12.8		110.4	3.8		92.8	6.7
PFNA	87.9	8.7		98.7	6.9		84.6	17.8
PFDA	90.7	5.8		85.7	18.2		107.8	6.8
PFHxS	84.6	10.8		104.8	14.7		102.6	12.8
PFHpS	96.8	12.8		107.9	15.0		92.8	18.0
FPOSA	116.8	16.8		112.5	14.2		107.6	12.4

### 2.3 与其他方法的比较

由于PFASs的种类较多,且血清基质较为复杂,一次检测血清中多种PFASs的方法并不多见^[20,21]^。Shafique等^[21]^基于气相色谱-串联质谱(GC-MS/MS)建立了血清中多种PFASs的分析方法,但所得到的回收率波动较大。Mazzoni等^[22]^开发了一种基于在线净化-液相色谱-串联质谱同时测定血清中两种全氟烷基磺酸盐(PFOS、PFHxS)、3种全氟烷基羧酸盐(PFOA、PFNA、PFDA)和一种全氟烷基磺酰胺(N-MeFOSA)的分析方法,6种目标物的回收率为84.0%~120.1%,LOD为0.16~0.34 ng/mL,但该方法在操作过程中需要两根色谱柱,且在线处理步骤较为复杂,样品用量大,因此推广性有限(见表5)。本方法可检测的PFASs种类多,准确度与精密度高,且试剂用量少,适用于人群中多种PFASs的快速分析测定。

**表5:** 本方法与其他文献方法的比较

Compounds	Analytical method	Detection time/min	Matrix	LODs/ (ng/mL)		Recoveries/ %		Reference
PFNA, PFDA, PFHpS, PFOSA, PFHxS, PFOA, PFOS	LLE-HPLC-MS/MS	20.0	serum	0.006-	0.020	84.6-	116.8	this work
PFOS, PFOA, PFOSA	LPE-LC-MS/MS	20.0	food	0.5-	1	82.2-	98.7	[20]
PFOS, PFHxS, PFOA, PFDA	SPE-LC-MS/MS	20.0	water	0.1-	0.5	89.0-	112.1	[21]
PFPeA, PFHxA, PFHpA, PFOA, PFNA, PFDA, PFUnDA	GC-MS/MS	24.0	serum	0.009-	0.2	71.6-	141.8	[22]
PFDoDA, PFBS, PFHxS, PFOS								
PFOS, PFHxS, PFOA, PFNA, PFDA, N-MeFOSA	TOF-LC-MS/MS	15.3	plasma	0.16-	0.34	84.0-	120.1	[23]

PFPeA: perfluoropentanoic acid; PFHxA: perfluorohexanoic acid; PFHpA: perfluoroheptanoic acid; PFUnDA: perfluoroundecanoic acid; PFDoDA: perfluorododecanoic acid; PFBS: perfluorobutanesulfonic acid; *N*-MeFOSA: *N*-methyl-perfluorooctanesulfonamide; LLE: liquid-liquid extraction; LPE: liquid-phase microextraction; TOF: time-of-flight.

### 2.4 孕妇血清中PFASs的暴露水平

456份孕妇血清样本中,暴露水平最高的是PFOA,中位数水平为19.4 ng/mL,检出率为100%;其次为PFOS,中位数水平为7.5 ng/mL,检出率为100%; PFHpS的中位数水平为5.62 ng/mL,检出率为98.5%; PFDA的中位数水平为2.01 ng/mL,检出率为97.6%; PFNA的中位数水平为1.92 ng/mL,检出率为100%; PFHxS的中位数水平为1.20 ng/mL,检出率为100%; PFOSA的中位数水平为0.55 ng/mL,检出率为97.4%。PFOA是血清中PFASs的最主要贡献者,其暴露水平最高的原因可能是其半衰期较长且使用广泛。研究发现,在食品包装、服装、家具、电器、炊具、化妆品等日常生活品中,PFOA的含量远高于其他PFASs^[24]^。此外,动物实验表明,血清中的白蛋白是PFASs的主要靶标,而PFOA与白蛋白的结合能力要强于其他PFASs,从而导致其在血清中的蓄积最高^[25]^。

### 2.5 新生儿神经行为评分

本研究中,379名新生儿的行为能力(满分12分)、被动肌张力(满分8分)、主动肌张力(满分8分)、原始反射(满分6分)、一般反应(满分6分)及NBNA总分(满分40分)分别为11.12±0.62、7.37±0.62、7.50±0.50、5.48±0.52、5.41±0.48和37.03±1.27分。如表6所示,性别分层分析结果显示,女性新生儿的行为能力(*P*=0.013)、主动肌张力(*P*=0.009)和原始反射(*P*=0.026)均高于男性新生儿。

**表6:** 新生儿神经行为测定结果(*n*=379)

NBNA score	All newborns	Male newborns	Female newborns	P^*^
Behavior	11.12±0.62	11.10±0.72	11.20±0.53	0.013
Passive muscle tone	7.37±0.62	7.40±0.62	7.35±0.63	0.210
Active muscle tone	7.50±0.50	7.40±0.62	7.52±0.50	0.009
Primitive reflexes	5.48±0.52	5.42±0.50	5.56±0.42	0.026
General assessment	5.41±0.48	5.42±0.41	5.41±0.50	0.248
Total score of NBNA	37.03±1.27	37.00±1.32	37.70±1.21	0.149

* *P*-values between male and female newborns.

### 2.6 孕期PFASs暴露对新生儿各功能区得分的影响

由于血清中PFASs的水平呈偏态分布,在随后的分析中将PFASs的水平值作对数转换,使其呈正态分布,再采用线性回归分析方法来探究孕期PFASs暴露与新生儿神经行为间的关系。

在对总人群的各个混杂因素进行调整后发现,孕期母体体内的PFOS每增加一个自然对数(ln)水平,新生儿的主动肌张力及一般反应分别降低0.36分(95%置信区间(CI): -0.64~-0.08, *P*=0.036)和0.34分(95% CI: -0.61~-0.07, *P*=0.040);孕期母体体内的PFNA每增加一个ln水平,新生儿的被动肌张力和NBNA总分分别降低0.38分(95%CI: -0.74~-0.01, *P*=0.042)和0.37分(95% CI: -0.68~-0.06, *P*=0.042); PFDA每增加一个ln水平,新生儿的行为能力降低0.28分(95% CI: -0.54~-0.01, *P*=0.044);而PFHxS每增加一个ln水平,新生儿的NBNA总分上升0.27分(95% CI: 0.05~0.48, *P*=0.038),详细结果如图2所示。美国^[26]^和日本^[27]^的两项研究使用贝利婴幼儿发展量表第3版(BSID-Ⅲ)来评估新生儿的神经行为发育,结果显示,孕妇产前暴露PFOS和PFNA与新生儿的认知行为下降和语言功能评分降低有关;虽然这两项研究与本次研究所使用的评分方法不一致,但均能说明孕期PFASs暴露对儿童神经行为发育有负面影响。

**图2:**
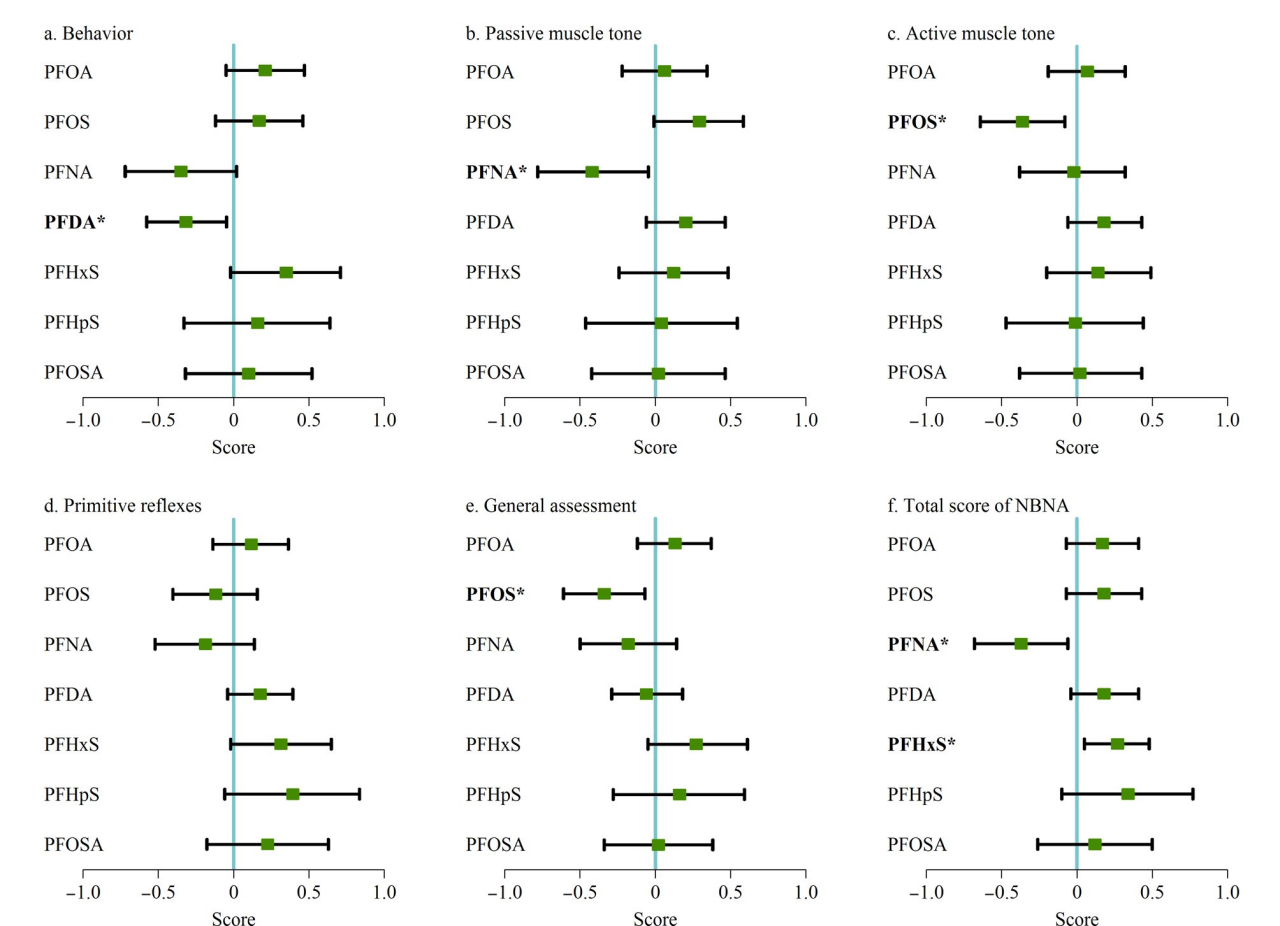
孕期PFASs暴露对新生儿各功能区得分的影响

### 2.7 孕期PFASs暴露对新生儿神经行为影响的性别差异

经性别分层后,孕期PFASs暴露对新生儿神经行为的影响如表7所示。在对各项混杂因素进行调整后,PFOS每增加一个ln水平,男性新生儿的主动肌张力及一般反应分别降低0.54分(95% CI: -0.73~-0.35)和0.50分(95% CI: -0.88~-0.13); PFNA每增加一个ln水平,男性新生儿的被动肌张力和NBNA总分分别降低0.67分(95% CI: -1.20~-0.14)和0.45分(95% CI: -0.91~-0.01); PFDA每增加一个ln水平,男性新生儿的行为能力降低0.44分(95% CI: -0.71~-0.17);而PFHxS每增加一个ln水平,男性新生儿的NBNA总分上升0.41分(95% CI: 0.02~0.80);同时,该性别分层研究未观察到PFASs与原始反射之间的统计关联。

**表7:** 孕期PFASs暴露对不同性别新生儿神经行为的影响

Compound	*β*(95% CI)^a^					
	Behavior	Passive muscle tone	Active muscle tone	Primitive reflexes	General assessment	Total score of NBNA
Male newborns (*n*=201)						
PFOA	0.20(-0.19, 0.59)	-0.07(-0.48, 0.32)	0.04(-0.32, 0.41)	0.01(-0.36, 0.38)	-0.02(-0.36, 0.32)	0.11(-0.25, 0.46)
PFOS	0.34(-0.07, 0.76)	0.20(-0.23, 0.65)	-0.54(0.73, -0.35)^*^	-0.04(-0.43, 0.36)	-0.50(-0.88, -0.13)^*^	0.30(-0.08, 0.68)
PFNA	-0.34(-0.85, 0.18)	-0.67(-1.20, -0.14)^*^	0.10(-0.38, 0.59)	-0.13(-0.62, 0.36)	-0.34(-0.79, 0.12)	-0.45(-0.91, -0.01)^*^
PFDA	-0.44(-0.71, -0.17)^*^	0.20(-0.19, 0.59)	0.08(-0.24, 0.43)	0.24(-0.12, 0.60)	0.17(-0.50, 0.16)	0.24(-0.11, 0.59)
PFHxS	0.46(-0.07, 0.98)	0.34(-0.23, 0.89)	0.52(-0.28, 1.30)	0.38(-0.13, 0.89)	0.29(-0.18, 0.77)	0.41(0.02, 0.80)^*^
PFHpS	0.25(-0.46, 0.96)	0.19(-0.55, 0.92)	0.40(-0.26, 1.06)	0.53(-0.14, 1.20)	-0.14(-0.78, 0.29)	0.49(-0.16, 1.15)
FPOSA	-0.04(-0.66, 0.59)	0.17(-0.48, 0.83)	0.41(-0.17, 1.00)	0.08(-0.52, 0.68)	-0.26(-0.82, 0.29)	0.13(-0.44, 0.71)
Female newborns (*n*=178)						
PFOA	0.24(-0.06, 0.54)	0.24(-0.12, 0.60)	0.18(-0.18, 0.54)	0.26(-0.07, 0.61)	-0.27(-0.51, -0.02)^*^	0.28(-0.02, 0.58)
PFOS	-0.05(-0.43, 0.34)	0.30(-0.10, 0.70)	-0.11(-0.51, 0.29)	-0.24(-0.61, 0.13)	0.19(-0.18, 0.56)	0.04(-0.29, 0.37)
PFNA	-0.20(0.70, 0.29)	0.10(-0.41, 0.59)	-0.01(-0.50, 0.49)	-0.13(-0.61, 0.34)	0.13(-0.34, 0.60)	-0.13(-0.54, 0.29)
PFDA	0.46(0.40, 0.52)^*^	0.19(-0.14, 0.53)	0.26(-0.07, 0.60)	0.04(-0.29, 0.36)	0.05(-0.26, 0.37)	0.06(-0.22, 0.35)
PFHxS	0.29(-0.19, 0.76)	0.05(-0.43, 0.53)	-0.30(-0.78, 0.17)	0.29(-0.17, 0.74)	0.35(-0.10, 0.80)	0.18(-0.22, 0.59)
PFHpS	0.12(-0.50, 0.74)	-0.01(-0.63, 0.61)	-0.29(0.90, 0.34)	0.42(-0.18, 1.02)	0.52(-0.07, 1.12)	0.22(-0.30, 0.74)
FPOSA	0.24(0.31, 0.80)	-0.07(-0.64, 0.49)	-0.31(-0.84, 0.24)	0.36(-0.18, 0.90)	0.40(-0.13, 0.92)	0.12(-0.35, 0.60)

* *P*<0.05; *β*: regression coefficient; a: per unit increase in the ln-transformed PFASs levels in serum. All models were adjusted for maternal age, pre-pregnancy BMI, living zone, mother’s schooling time, annual household income, parity, residential building type and average living space.

在对各项混杂因素进行调整后,孕期母体PFASs暴露对女性新生儿神经行为的影响较小。如表7所示,仅观察到PFOA与一般反应之间呈负相关关系(回归系数(regression coefficient, *β*)=-0.27; 95% CI: -0.51~-0.02),而PFDA与行为能力之间呈正相关关系(*β*=0.46; 95% CI: 0.40~0.52)。这一结果与丹麦的一项出生队列研究类似,即PFASs对男性新生儿身长、体重、认知发育等方面的影响大于女性新生儿^[28]^。原因可能是孕期PFASs暴露会干扰母体甲状腺相关激素(如促甲状腺激素、游离三碘甲状腺原氨酸)的合成及转运,甲状腺相关激素的分泌又会影响儿童的神经行为发育;而由于性别差异,不同性别新生儿对甲状腺激素的敏感度不同,导致PFASs对神经行为的影响存在性别差异^[29]^。

## 3 结论

本研究建立了测定孕妇血清中7种PFASs的高效液相色谱-串联质谱法,该方法检出限低,回收率及精密度良好,且样品用量少。将该方法应用于已建立的出生队列,结果显示,孕妇血清中PFOA的暴露水平最高;同时,我们随访并测定了子代新生儿的神经行为评分,结果证实,孕期PFOS、PFNA和PFDA的暴露与新生儿NBNA的评分呈负相关。对子代新生儿进行性别分层分析后发现,孕期PFASs暴露对男性新生儿的神经行为影响更为明显。由于本研究只采集了孕妇孕晚期的血清样本,未能探索孕早期及孕中期PFASs暴露对新生儿神经发育的影响,无法确定孕妇暴露PFASs的敏感窗口期。今后可通过动物模拟实验,探究PFASs神经发育毒性的敏感窗口期及其致病机制。
